# Mesenchymal stromal cells for cutaneous wound healing in a rabbit model: pre-clinical study applicable in the pediatric surgical setting

**DOI:** 10.1186/s12967-015-0580-3

**Published:** 2015-07-08

**Authors:** Gloria Pelizzo, Maria Antonietta Avanzini, Antonia Icaro Cornaglia, Monica Osti, Piero Romano, Luigi Avolio, Rita Maccario, Massimo Dominici, Annalisa De Silvestri, Erika Andreatta, Federico Costanzo, Melissa Mantelli, Daniela Ingo, Serena Piccinno, Valeria Calcaterra

**Affiliations:** Pediatric Surgery Unit, Fondazione IRCCS Policlinico San Matteo and University of Pavia, 27100 Pavia, Italy; Immunology and Transplantation Laboratory/Cell Factory/Pediatric Hematology/Oncology Department, Fondazione IRCCS Policlinico San Matteo, Pavia, Italy; Histology and Embryology Unit, Department of Public Health, Experimental Medicine and Forensic, University of Pavia, Pavia, Italy; Laboratory of Cellular Therapies, Department of Medical and Surgical Sciences for Children and Adults, University Hospital of Modena and Reggio, Emilia, Italy; Biometry and Clinical Epidemiology Unit, Scientific Direction, Fondazione IRCCS Policlinico San Matteo, Pavia, Italy; Pediatric Unit, Fondazione IRCCS Policlinico San Matteo and University of Pavia, Pavia, Italy

**Keywords:** Mesenchymal stromal cells, Cutaneous wounds, Adipose, Bone marrow, Pediatric surgery, Regenerative medicine

## Abstract

**Objective:**

Mesenchymal stromal cells (MSCs) expanded in vitro have been proposed as a potential therapy for congenital or acquired skin defects in pediatrics. The aim of this pre-clinical study was to investigate the effects of intradermal injections of MSC in experimental cutaneous wound repair comparing allogeneic and autologous adipose stem cells (ASCs) and autologous bone marrow-mesenchymal stromal cells (BM-MSCs).

**Methods:**

Mesenchymal stromal cells were in vitro expanded from adipose and BM tissues of young female New Zealand rabbits. MSCs were characterized for plastic adhesion, surface markers, proliferation and differentiation capacity. When an adequate number of cells (ASCs 10 × 10^6^ and BM-MSCs 3 × 10^6^, because of their low rate of proliferation) was reached, two skin wounds were surgically induced in each animal. The first was topically treated with cell infusions, the second was used as a control. The intradermal inoculation included autologous or allogeneic ASCs or autologous BM-MSCs. For histological examination, animals were sacrificed and wounds were harvested after 11 and 21 days of treatment.

**Results:**

Rabbit ASCs were isolated and expanded in vitro with relative abundance, cells expressed typical surface markers (CD49e, CD90 and CD29). Topically, ASC inoculation provided more rapid wound healing than BM-MSCs and controls. Improved re-epithelization, reduced inflammatory infiltration and increased collagen deposition were observed in biopsies from wounds treated with ASCs, with the best result in the autologous setting. ASCs also improved restoration of skin architecture during wound healing.

**Conclusion:**

The use of ASCs may offer a promising solution to treat extended wounds. Pre-clinical studies are however necessary to validate the best skin regeneration technique, which could be used in pediatric surgical translational research.

## Background

Renewal and maintenance of normal skin depend on a pool of endogenous progenitors. After skin injury, a series of coordinated events occur, including bleeding and coagulation, acute inflammation, cell migration, proliferation, differentiation, angiogenesis, re-epithelialization, and synthesis as well as remodelling of the extracellular matrix. These complex events involve three overlapping phases: inflammation, proliferation and remodelling [1–4]. In the normal healing process, all of the above stages function sequentially and for a specific amount of time. However, interference during the wound healing process may prolong one or more of these phases leading to delayed or incomplete wound healing.

Recent advances in stem cell therapy for tissue engineering approaches have provided promising data on wound repair and tissue regeneration. Mesenchymal stromal cells (MSCs) expanded in vitro have been proposed as a potential therapy to enhance cutaneous wound healing [5–11]. It has been described that MSCs may attenuate the inflammatory response by influencing the wound’s ability to progress beyond the inflammatory phase and not regress to a chronic wound state [12, 13]. MSCs are multipotent cells that can differentiate into multiple tissue-forming cell lineages, such as osteoblasts, adipocytes, chondrocytes, tenocytes, and myocytes. In addition, MSCs regulate immune and inflammatory responses. These multipotent cells with innate self-renewal capacity can be in vitro expanded without losing their differentiation potential. MSCs have been isolated from various tissues such as bone marrow (BM), umbilical cord blood, skeletal muscle and brain. Additionally, they can be easily obtained in large quantities with minimal invasiveness from adipose tissue, the so called adipose stem cells (ASCs) [14, 15]. Although BM-MSCs and ASCs share many biological features, there are some differences between these distinct MSC populations. For instance ASCs show a higher proliferative capacity and retain differentiation potential for a longer period in culture compared with human BM-MSCs. Moreover it has been demonstrated that human ASCs support hematopoiesis both in vitro and in vivo more efficiently than human BM-MSCs, on the other hand it has been suggested that BM-MSCs evoke less inflammation and thrombogenesis than ASC [16].

The therapeutic effects of MSCs have already been demonstrated in preclinical and clinical studies in adults [6]. Their strong capacity to proliferate and differentiate and their immunomodulatory effects support their potential use in safe regenerative medicine approaches also in pediatric patients [17]. In the presence of extensive and disfiguring congenital pathologies, impaired wound healing remains a challenge and causes debilitating effects with tremendous suffering. This is the case with giant congenital melanocytic nevi, where complete surgical removal is difficult to achieve because of the lack of available skin to graft over the resultant defects [18, 19] or following acquired skin lesions such as burn wounds or tissue destruction following surgery or trauma in which skin damage usually never fully recovers [20, 21].

In these cases, ASCs or BM-MSCs represent tools which could potentially provide mechanical and humoral support for skin regeneration for better functional and cosmetic results. However, the use of MSC therapy to improve wound healing is limited as a widely used and proven cell source is not rapidly available after injury. In fact, the time necessary to ex vivo expand sufficient autologous cell numbers is extensive. The alternative is to use allogeneic cells that can be pre-expanded and characterised, and thus ready for use in the initial stages of injury.

The aim of this pre-clinical study was to investigate the advantage of using autologous ASCs over BM-MSCs, evaluating the effects of intradermal injections in experimental cutaneous wounds. Our purpose was to obtain a model which could be translated to the pediatric surgical setting. Considering the technical difficulties in obtaining adipose tissue in the pediatric age compared to the adult age and knowledge that age negatively impacts the biologic features of MSCs [22], we used young animals. Allogenic ASCs were also considered as an alternative source when autologous ASC isolation would be difficult, such as in neonates or young infants.

## Methods

### Adipose tissue and bone marrow harvest

Healthy young female New Zealand rabbits (n = 27, 3 months old, median 3.5 kg weight) [23], were used as an animal model. The experimental protocol was approved by the National Animal Care and Ethics Committee and conducted in accordance with Italian and European legislation (D.lgs. 116/92, European Directives 86/609/EE and 2010-63UE for the protection of animals used in scientific and experimental studies).

After overnight fasting, experimental animals were premedicated with an intramuscular midazolam injection (1 mg/kg). Under general anesthesia using Zoletil 0.4 ml/kg (Virbac, Milano, Italy), and after local anesthesia with levo-bupivacaine 0.25% or ropivacaine 0.2% (2 ml/cm wound), a 2 cm longitudinal incision was performed in the inguinal area in order to harvest adipose panicle (lipectomy), while BM was harvested by aspiration from the femoral medullary cavities.

Local anesthesia with levo-bupivacaine 0.25% or ropivacaine 0.2% (2 ml/cm wound) were repeated for pain management. Subcutaneous Enrofloxacin (0.1 ml/2 kg/day for 3 days—Bayer, Milano, Italy) and Meloxicam (0.3 mg/kg/day for 3 days—Boehringer Ingelheim, Milano, Italy) were subsequently administered.

### Isolation and culture of ASCs and BM-MSCs

Inguinal fat pads were placed in sterile phosphate-buffered saline (PBS, Euroclone, Milan, Italy) with gentamicin, minced manually and digested with 0.0075% type II collagenase (3 mg/ml, Sigma-Aldrich, St Louis, MO, USA) in Dulbecco’s modified Eagle Medium (DMEM, Gibco, Invitrogen, Monza, Italy) for 20 min at 37°C with gentle agitation. The stromal vascular fraction (SVF), containing ASCs, was suspended in DMEM +10% Fetal Bovine Serum (FBS, Euroclone), in order to inhibit enzyme activity. The specimen was then filtered through a 100 mm sterile nylon mesh filter (Millipore, Darmstadt, Germany), and centrifuged at 1,200 rpm for 10 min. The resultant pellet was suspended and counted with 0.4% Trypan blue (Sigma-Aldrich). Cells were subsequently plated in culture flasks (Corning Costar, Amsterdam, The Netherlands) at a density of 160,000/cm^2^ in αMEM (Gibco, Invitrogen) containing 10% FBS (Euroclone) and 1% antibiotic–antimycotic (Sigma-Aldrich) at 37°C, 5% CO_2_ in a humidified atmosphere.

Mononuclear cells (MNC) were isolated from 1 ml of BM aspirate, by density gradient centrifugation (Ficoll 1.077 g/ml; Lympholyte, Cedarlane Laboratories Ltd., The Netherlands) counted and plated in 75 or 175 cm^2^ tissue culture flasks at a density of 160,000/cm^2^ in αMEM supplemented with 10% FBS (Euroclone) and 1% antibiotic–antimycotic (Sigma-Aldrich). Cultures were maintained at 37°C, 5% CO_2_ in a humidified atmosphere. After 48-h, non-adherent cells were removed and culture medium was replaced twice a week. After reaching ≥80% confluence, MSCs were harvested using Trypsin–EDTA (Lonza, Copenhagen, Denmark), and propagated at 4,000 cells/cm^2^.

ASC were expanded until passage (P)4, while BM-MSC were expanded until P3 because of their low rate of proliferation. At each passage, viable cells were counted using 0.1% eosin and culture supernatants were tested for sterility.

### Immunophenotype

Rabbit ASCs and BM-MSCs were characterized by flow-cytometry. Fluorescein isothiocyanate (FITC)- or phycoerythrin (PE)-conjugated monoclonal antibodies (BD PharMingen, San Diego, CA, USA) anti-human CD29, anti-rat CD45, anti-human CD49e, anti-human CD10 and anti-rat CD90 cross-reactive against rabbit were used as described [24]. Appropriate, isotype-matched, irrelevant fluorochrome-conjugated antibodies were used as controls.

### Differentiation assays

ASCs and BM-MSCs were evaluated for their ability to differentiate into osteoblasts and adipocytes, as previously described [25].

The osteogenic differentiation capacity of ASCs and BM-MSCs was assessed at P2–4 by incubating cells with αMEM, 10% FBS, 1% gentamicin, supplemented with 10^−7^ M dexamethasone (Sigma-Aldrich St Louis, MO, USA), 50 mg/ml l-ascorbic acid (Sigma-Aldrich). Starting from day +7 of culture, 5 mM β-glycerol phosphate (Sigma-Aldrich) was added to the medium. Adipogenic differentiation was evaluated at P2–4 by incubating cells with αMEM, 10% FBS, 1% gentamicin supplemented with 10^−7^ M dexamethasone, 50 mg/ml l-ascorbic acid, 100 mg/ml insulin, 50 mM isobutyl methylxanthine (Sigma-Aldrich), 0.5 mM indomethacin (MP Biomedica, Illkirch, France) and 5 mM β-glycerol phosphate. Both osteogenic and adipogenic cultures were incubated for 21 days before evaluating differentiation. To detect osteogenic differentiation, cells were stained for alkaline phosphatase (AP) activity using Fast Blue (Sigma-Aldrich) and, for calcium deposition, by Alizarin Red S staining (Sigma-Aldrich). Adipogenic differentiation was evaluated through the morphological appearance of fat droplets stained with Oil Red O (Bio Optica, Milan, Italy).

### Semi-solid clonogenic assay

A suspension of 200,000 cells in D-MEM low glucose (Gibco), 0.9% methylcellulose (StemCell Technologies, Milan, Italy), 30% (vol/vol) FBS (StemCell Technologies) was plated in six-well plates. After 4 weeks incubation at 37°C in a humidified atmosphere with 5% CO_2_, the plates were examined with contrast phase microscopy (4×) for the formation of colonies with an inverted microscope (Leitz, Wetzlar, Germany). A malignant transformed rat MSC line, obtained in our laboratory, was used as a positive control.

### Senescence assay

Rabbit ASCs and BM-MSCs were maintained in culture until reaching senescence. They were closely monitored during senescence for up to 8–12 weeks before interrupting the cultures, in order to reveal any change in morphology and/or proliferation rate. MSC senescence was assessed by staining with the β-galactosidase (SA-β-gal) staining kit (Cell Signaling Technology, Danvers, MA, USA), according to the manufacturer’s instructions, and evaluated by direct-light microscopy.

### Rabbit cutaneous wound model

When an adequate number of cells was reached, the skin wounds were surgically induced. After overnight fasting, experimental animals was premedicated with intramuscular midazolam (1 mg/kg). Under general anesthesia using Zoletil 0.4 ml/kg, and after local anesthesia with levo-bupivacaine 0.25% or ropivacaine 0.2% (2 ml/cm wound), two identical full thickness 2 × 2 cm wounds were created on the back of each rabbit, at a distance of more than 2 cm from each other. One lesion was used as a control. After creation of the wounds, local anesthesia with levo-bupivacaine 0.25% or ropivacaine 0.2% (2 ml/cm wound) was repeated. Subcutaneous Enrofloxacin (0.1 ml/2 kg/day for 3 days) and Meloxicam (0.3 mg/kg/day for 3 days) were subsequently administered.

### Intradermal injection of ASCs and BM-MSCs

Within 5 min from the wound lesion establishment, autologous or allogeneic ASCs or autologous BM-MSCs in 3 ml of saline, 2% rabbit albumin (Sigma-Aldrich) were directly injected into the wound bed of the first lesion. The protocol called for infusions of 10 × 10^6^ cells into the wound bed. An injection of 3 ml of saline, 2% rabbit albumin solution was used as a control in the second lesion. Rabbits did not receive any immune suppression. General conditions of the animals and wound healing were monitored daily. Wounds were photographed using a digital camera.

**Figure 1:**
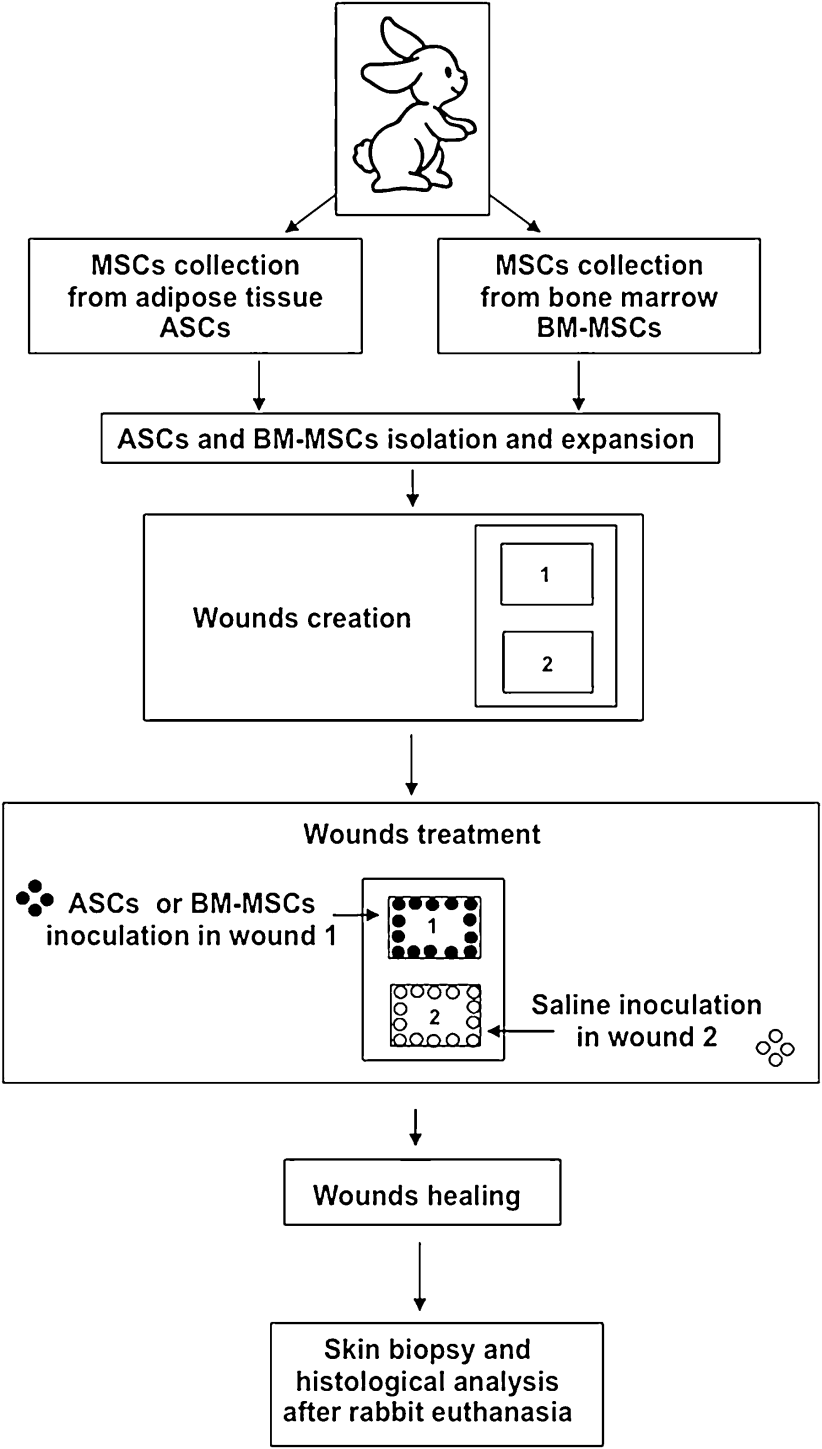
Experimental design. Rabbit ASCs and BM-MSCs were collected, isolated and expanded. As soon as an adequate number of in vitro expanded MSCs was reached, two wound lesions (wound 1 and wound 2) were created. Within 5 min from the wound lesion establishment, autologous or allogeneic ASCs or autologous BM-MSCs were directly injected into the wound bed 1. Into wound bed 2, 3 ml of saline 2% rabbit albumin solution was injected as a control. Wound healing was monitored daily. After rabbit euthanasia, biopsies of the regenerated tissues were collected, using dermal biopsy punches, for histological examination.

For the experimental outline see Figure 1.

### Histological and immunohistochemical analyses

Rabbits were euthanized with a bolus of Pentobarbital 100 mg/kg I.V. For histological examination, wounds were harvested after 11 (12 rabbits) and 21 days (15 rabbits) of treatment. The regenerated tissue biopsies were collected using dermal biopsy punches. The samples were bisected along the length of the wound, fixed in 4% neutral buffered formalin for 48 h, dehydrated with a gradient alcohol series, cleared in xylene and eventually embedded in paraffin. Sections (8 μm) were obtained using a Leitz microtome and prepared for histology. All stained slides were examined under a Axiophot Zeiss light microscope (Oberkochen, Germany) equipped with a digital camera.

To compare the re-epithelization rate and the amount of inflammatory infiltration, tissue sections were stained with haematoxylin and eosin (H&E). To assess the degree of collagen synthesis, Mallory’s trichrome stain was used, while immunohistochemical evaluation of proliferation was performed by anti-Proliferating Cell Nuclear Antigen (PCNA) antibody (Dako, Glostrup, Denmark). In this latter case, tissue sections were deparaffinized, hydrated and pretreated for antigen retrieval using a pressure cooker in 10 mM citrate buffer. Endogenous peroxidases were quenched with 3% hydrogen peroxide for 15 min. Nonspecific antibody binding was blocked by incubation with a protein blocker (Dako). The sections were incubated with 1:400 anti-PCNA antibody or bovine serum albumin as a negative control for 30 min at 45°C, then incubated with horseradish peroxidase conjugated anti-mouse immunoglobulin (Dako) for 15 min at 45°C, followed by a 5 min incubation with 3,3′-diaminobenzidine (DAB; Dako). Finally, the sections were mounted and examined under a light microscope [26].

The re-epithelization rate defined as the presence of differentiated multi-layered epithelium, the amount of inflammatory infiltration defined as the presence of inflammatory cells [27], the degree of collagen defined as collagen content in granulation tissue and cellular proliferation, defined as PCNA positive nuclei/mm of epidermis or /mm^2^ of dermis were assessed using a semi-quantitative scale, with scores ranging from 0 to 3 as reported in Table 1. The 39 biopsies were examined by two independent operators.

**Table 1:** Semi-quantitative scale for the histological parameters

Score	0	1	2	3
Re-epithelization rate	Early signs of re-epithelization	Regenerated mono-layered epithelium	Regenerated multi-layered epithelium	Differentiated multi-layered epithelium (*stratum corneum*)
Inflammatory infiltrate (white cells)	None	Few	Fair	Rich
Collagen content in granulation tissue	None	Poor	Moderate	High
PCNA in epidermis and dermis (positive nuclei/mm of epidermis or mm^2^ of dermis)	0	1–9	10–30	>30

### Statistical analysis

Quantitative data and the histological score were described as the median and interquartile range (IQR: 25th–75th centile) and compared by fitting multivariable ordinal logistic regression models with robust standard errors to take into account the clustered structure of the data. Groups (using controls as a reference) and times (using 11 days as a reference) were included in the models as independent variables.

Statistical significance was defined as a p value <0.05. Since no time effect was observed we reported only the p values (obtained in the multivariable ordinal logistic regression models i.e. corrected for time) relative to group comparisons. Data analysis was performed with the STATA statistical package (release 13.1.2012, Stata Corporation, College Station, TX, USA).

## Results

### ASC and BM-MSC expansion rate and intradermal inoculation

ASCs were isolated from all rabbits and expanded to P4, while BM-MSCs were isolated and expanded to P3 in 4 out of 27 rabbits. The expansion rate, defined as the calculated cell count (ccc), for BM-MSC was significantly lower (p = 0.028) than ASCs, with a mean ccc value of 6.59 × 10^6^ ± 4.85 × 10^6^ at P3 and 199 × 10^6^ ± 263 × 10^6^ at P4, respectively. Moreover the time to reach confluence was longer for BM-MSC than ASC (median 13 days, range 8–21 day; median 6.5 days, range 3–11 day, respectively).

Due to this low rate of in vitro expansion and restrictions on the length of time to ethically and legally house the rabbits, we were only able to expand a lower number of BM-MSC. Even though, the original protocol called for infusions of 10 × 10^6^ cells into the wound bed, we decided to proceed with 3 × 10^6^ of BM-MSCs only in the autologous setting.

In order to compare results between ASC and BM-MSCs infusion, four rabbits were treated with a comparable number of ASCs.

### ASC and BM-MSC characterization

**Figure 2:**
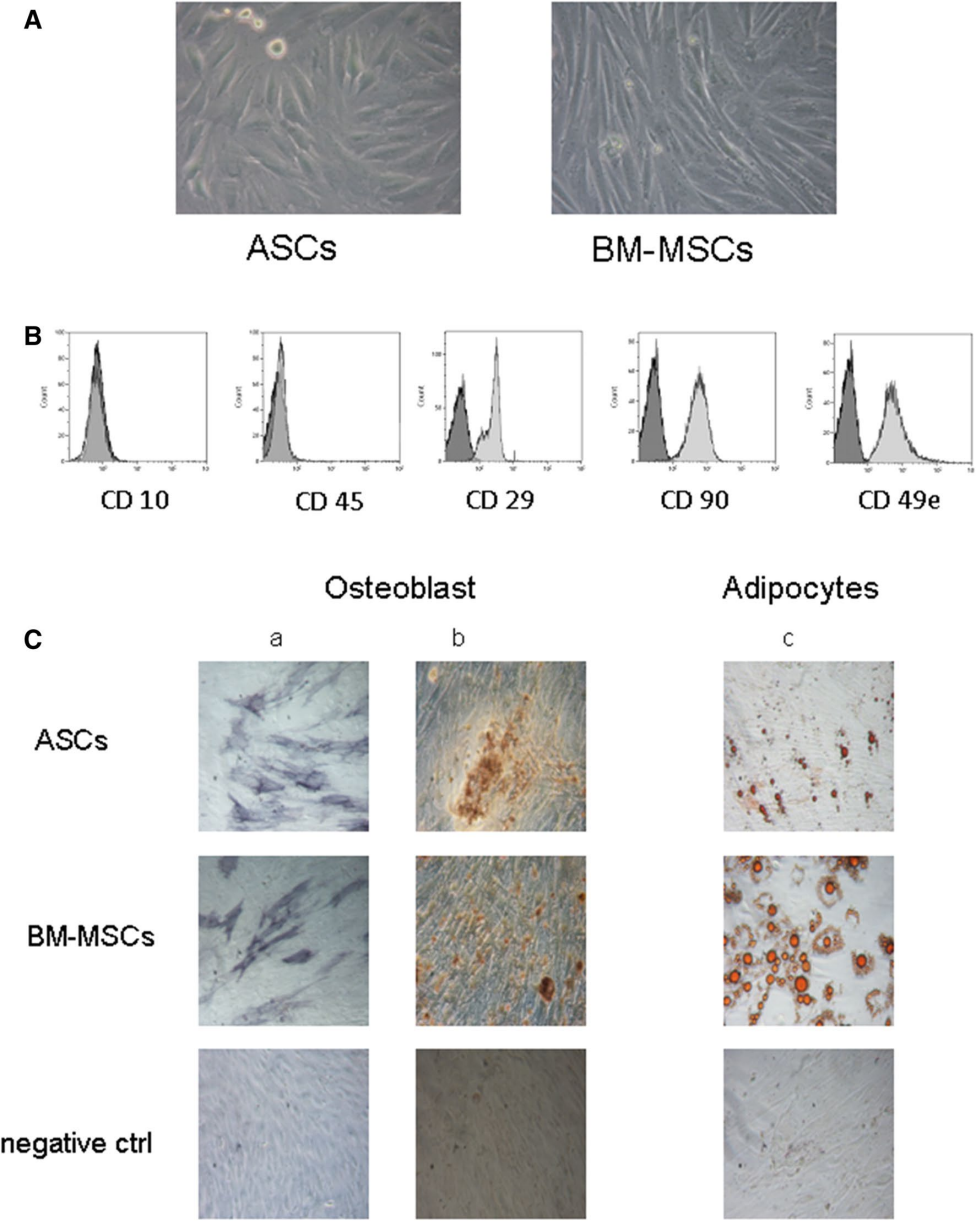
Characterization of rabbit MSCs. **A** Morphology of bone marrow and adipose-derived mesenchymal stromal cells obtained from one rabbit. MSCs from both sources display the characteristic spindle-shaped morphology. Magnification ×4. **B** Immunophenotype of culture-expanded ASCs obtained from one representative rabbit. ASCs were positive for CD90, CD29 and CD49e and negative for CD45 and CD10, as reported [20]. The immunophenotype of BM-MSCs was superimposable. **C** Osteogenic and adipogenic differentiation capacity of BM-MSCs and ASC. Differentiation into osteoblasts was demonstrated by the histological detection of Alkaline phosphatase activity (*a*) and calcium depositions positive for Alizarin Red (*b*); magnification ×20. Differentiation into adipocytes was revealed by the formation of lipid droplets stained with Oil Red O (*c*); magnification ×20. Non differentiated cells (negative ctrl) are reported.

ASCs and BM-MSCs showed the typical spindle shape morphology (Figure 2, Panel A) and they resulted positive for CD49e, CD90 and CD29, while they were negative for CD45 and CD10 (Figure 2, Panel B for in vitro-expanded ASCs) as reported by Piccinno et al. [21].

Both ASCs and BM-MSCs exhibited the capacity to differentiate in vitro to osteoblasts and adipocytes as confirmed by histological staining. Microscopic examination of stained cells demonstrated the presence of mineralization nodules and fat droplets (Figure 2, Panel C). Both types of mesenchymal progenitors ceased their growth at variable passages and entered into the senescence phase (data not shown), moreover the three lots tested were not capable of anchorage independent growth when cultured in a semisolid medium, indirectly confirming the lack of oncogenic transformation.

### Macroscopic aspect of the wound healing

**Figure 3:**
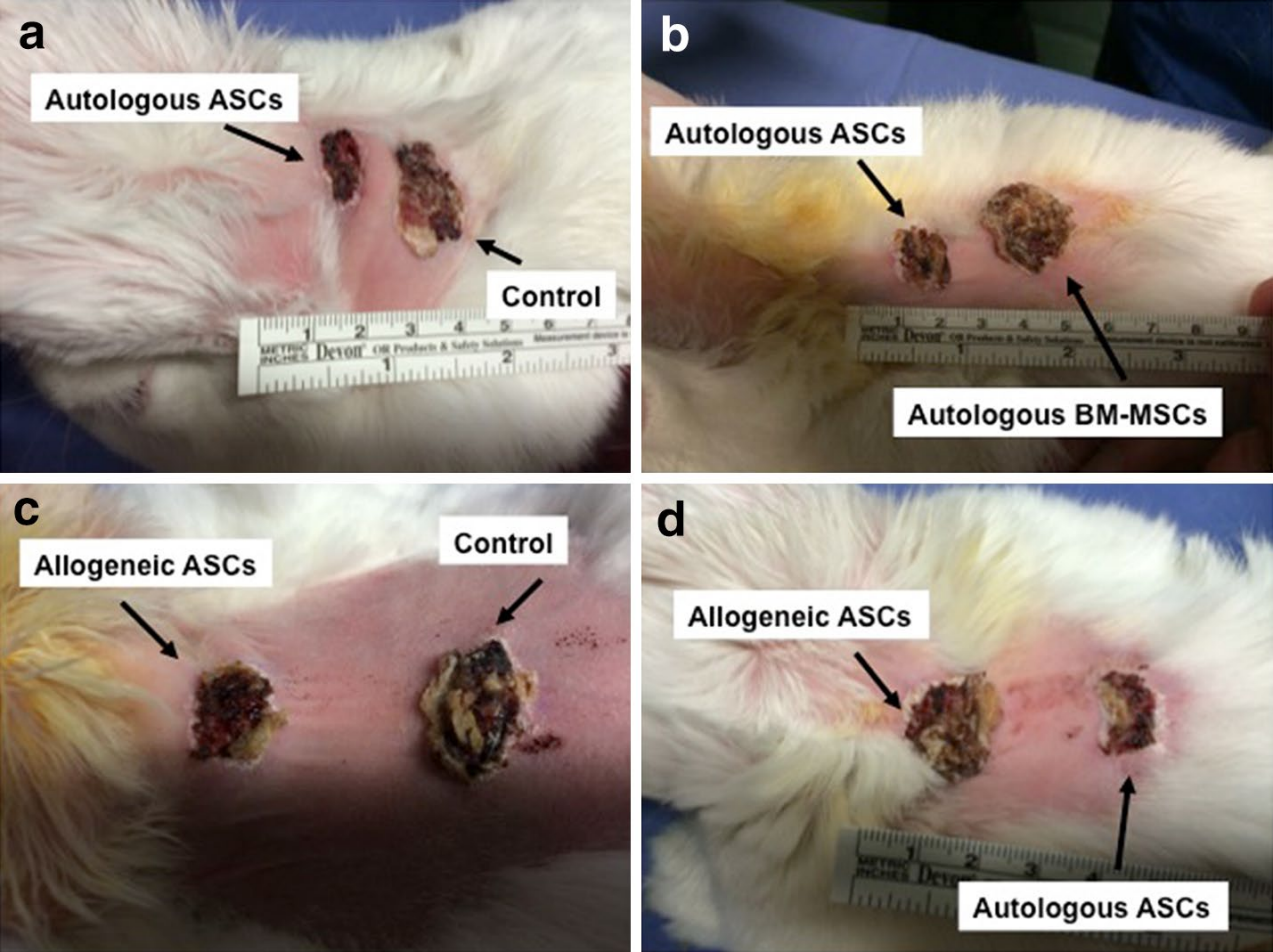
Skin regeneration at day 11 after autologous ASC injection vs control (**a**), autologous ASCs *vs* autologous BM-MSCs (**b**), allogeneic ASCs vs control (**c**), allogeneic ASCs *vs* autologous ASCs (**d**).

All wounds healed without severe infection and typical scars formed at the site of each wound. With the macroscopic analysis, autologous ASC inoculation induced a more rapid and more complete wound healing process when compared with the other experimental conditions (Figure 3).

### Histological results

#### Semi-quantitative analysis of histological parameters

Semi-quantitative assessment of the regenerated epithelial layer features, inflammatory infiltrate extent, collagen deposition and PCNA-positive nuclei in treated and control wounds, at 11 and 21 days, is reported in Table 2. In our study, the best and worst re-epithelization rates were noted after autologous ASC and autologous BM-MSC inoculation, respectively. Since no time effect was noted, we only reported the p values (obtained in the multivariable ordinal logistic regression models i.e. corrected for time) relative to group comparisons (Table 2).

**Table 2:** Semi-quantitative assessment of the regenerated epithelial layer features, inflammatory infiltrate extent, collagen deposition and PCNA-positive nuclei in differently treated wounds at 11 and 21 days

		11 days	21 days	p values vs ctrl
Autologous ASCs (n = 14)	Epithelial regeneration	3 (2–3)	3 (3–3)	p < 0.001
	Inflammatory infiltrate	2 (1–2)	1 (1–2)	p < 0.001
	Collagen deposition	2 (2–3)	3 (2–3)	p < 0.001
	PCNA-positive nuclei			
	Epidermis	3 (1–3)	3 (2–3)	p < 0.001
	Dermis	2 (1–3)	2 (2–3)	
Allogeneic ASCs (n = 9)	Epithelial regeneration	2 (2–2)	1 (1–1)	p < 0.001
	Inflammatory infiltrate	2 (2–3)	2 (2–3)	p = 0.039
	Collagen deposition	2 (2–2)	2 (2–2)	p < 0.001
	PCNA-positive nuclei			
	Epidermis	2 (1–3)	2 (1–2)	p < 0.01
	Dermis	2 (2–3)	2(2–3)	
BM-MSCs (n = 4)	Epithelial regeneration	1 (1–2)	1 (1–1)	p < 0.001
	Inflammatory infiltrate	3 (2–3)	3 (3–3)	p = 0.99
	Collagen deposition	1 (0–2)	1 (1–1)	p = 0.43
	PCNA-positive nuclei			
	Epidermis	1 (1–2)	2 (2–2)	p < 0.01
	Dermis	2 (2–3)	2 (2–2)	
Control wounds (n = 27)	Epithelial regeneration	0 (0–1)	1 (0–1)	
	Inflammatory infiltrate	3 (3–3)	3 (2–3)	
	Collagen deposition	0 (0–1)	1 (1–2)	
	PCNA-positive nuclei			
	Epidermis	0 (0–1)	3 (2–3)	
	Dermis	0 (0–1)	3 (3–3)	

Since no time effect was observed, only the p values (obtained in the multivariable ordinal logistic regression models i.e. corrected for time) relative to group comparisons are reported. Results are reported as the median and range score (Table 1).

*ASCs*  adipose derived stem cells, *BM-MSCs* bone marrow derived stem cells.

When MSC inoculation numbers were considered, histological results were not different in wounds treated with 10 × 10^6^ or 3 × 10^6^ autologous ASCs (epithelial regeneration p = 0.32; inflammatory infiltrate p = 0.34; collagen deposition p = 0.38; PCNA-positive nuclei-epidermis p = 0.26, dermis p = 0.68).

When different sources were considered, ASC-treated wounds exhibited better regeneration of epithelial layers (p < 0.001), collagen deposition (p < 0.001) and PCNA-positive nuclei in epithelial regenerated epidermis (p < 0.001) compared to BM-MSC treated lesions.

#### Re-epithelization, collagen deposition and cellular proliferation

**Figure 4:**
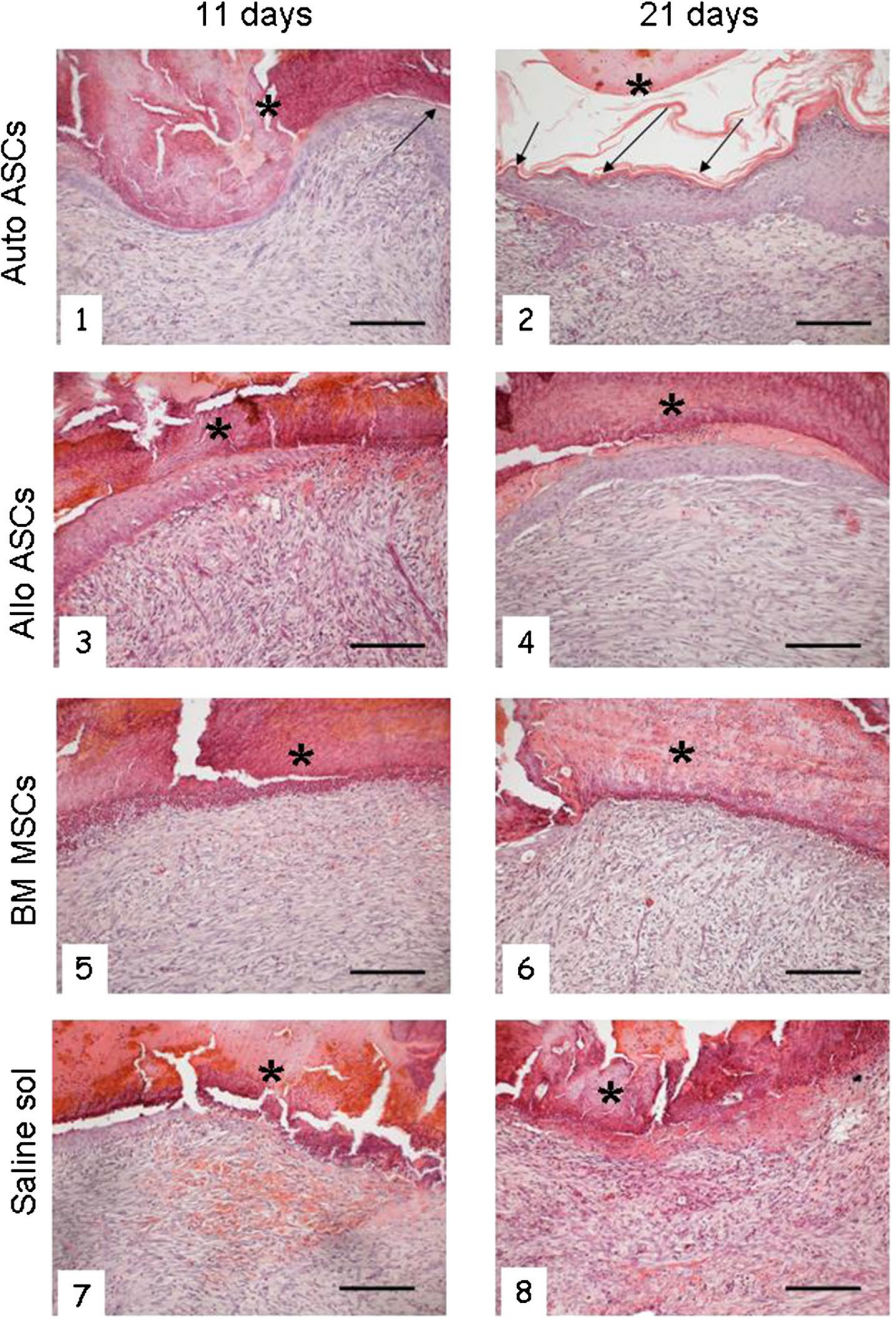
Histological aspect of the skin regeneration after 11 and 21 days of treatment. *Panel 1* after 11 days treatment with autologous ASCs, the epidermis was entirely regenerated and almost completely differentiated, with a thin *stratum corneum* (*arrow*) and some shedding of necrotic material (*asterisk*). The dermis contains a small number of inflammatory cells and some small blood vessels. *Panel 2* after 21 days treatment, the skin was completely regenerated, with a perfectly differentiated epidermis. *Panel* *3* allogeneic ASC-treated wounds after 11 days display large areas devoid of epithelium or with a mono-layered or thin epidermis. *Panel 4* after 21 days the epithelium was multi-layered, but no *stratum corneum* is evident. Connective tissue showed a small amount of inflammatory infiltration, with several dilated vessels. *Panels 5*, *6* the wounds inoculated with BM-MSCs show large areas devoid of epithelium after 11 days, as well as 21 days of treatment. *Panels* *7*, *8* only early signs of re-epithelization are detectable in saline-treated samples; in correspondence with the de-epithelized area the connective tissue is infiltrated by numerous inflammatory cells (granulation tissue) and many vessels appear dilated. Haematoxilin and Eosin staining. *Scale bar* 200 μm; original mag. ×100.

At 11 days, autologous ASC-treated wounds showed a more advanced re-epithelization in comparison with wounds treated under all other experimental conditions. Beneath the necrotic material, the epidermis was entirely regenerated and almost completely differentiated, with a thin *stratum corneum*. Only the central area of the wound had a single layer of epithelium. The dermis contained a small number of inflammatory cells; some small blood vessels, presumably newly formed, were evident, with only slight signs of edema (Figure 4, Panel 1). After 21 days of treatment, the skin was completely regenerated, with a perfectly differentiated epidermis (Figure 4, Panel 2).

Allogeneic ASC-treated wounds did not develop an entirely re-formed skin layer: a continuous mono-layer of epithelium was observed in most of the samples (Figure 4, Panel 3). At 21 days, the epithelium was multi-layered, but the central area of the wound never developed a *stratum corneum* (Figure 4, Panel 4). Connective tissue showed small amounts of inflammatory infiltration, with several dilated vessels.

The wounds inoculated with BM -MSCs showed, in most cases, a small area devoid of epithelium (Figure 4, Panels 5 and 6), while the saline-treated wounds never developed a continuous epidermis (Figure 4, Panels 7 and 8).

**Figure 5:**
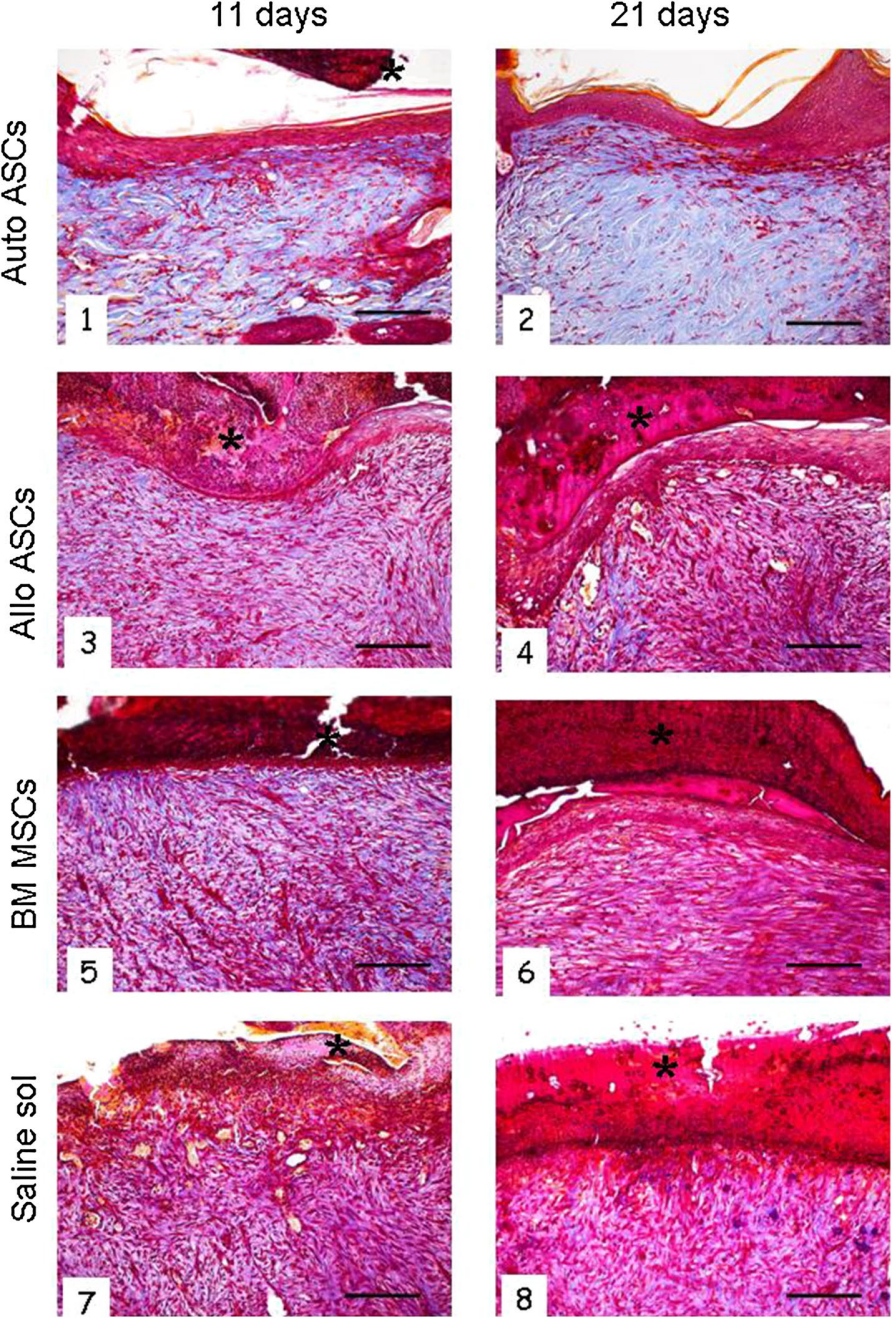
Histological evaluation of collagen deposition at 11 and 21 days after different MSC treatments. *Panels 1*, *2* blue-stained collagen was clearly detectable in autologous ASC-treated areas, where collagen is arranged in thick bundles of fibres. Allogeneic ASC- (*Panels* *3*, *4*) and BM-MSC- treated wounds (*Panels* *5*, *6*) show a slightly higher blue staining density than the saline-treated wounds (*Panels* *7*, *8*). Eschar (*asterisk*). Mallory trichrome-staining. *Scale bar* 200 μm; original mag. ×100.

In the Mallory trichrome-stained sections (Figure 5), all MSC-treated wounds showed higher blue staining density than the saline-treated wounds, revealing new collagen deposition. The amount of collagen visibly increased in autologous ASC-treated wounds where collagen was arranged in thick bundles of fibres (Figure 5, Panels 1 and 2). No differences were observed after 11 or 21 days of treatment.

**Figure 6:**
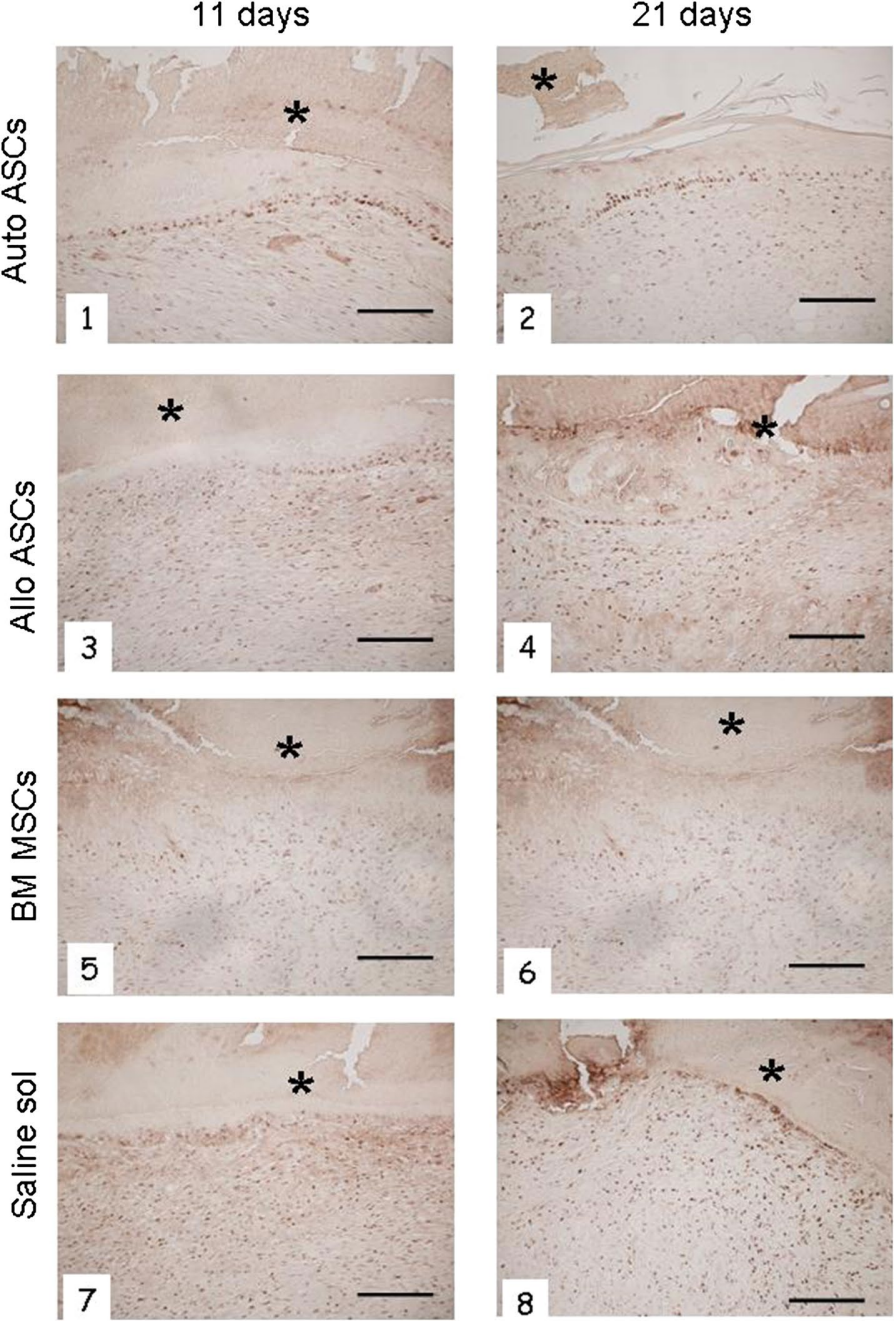
Histological evaluation of proliferative cellular nuclear antigen (PCNA)-positive nuclei at 11 and 21 days after different MSC treatments. *Panels* *1*, *2* autologous ASC-treated wounds show a high number of PCNA-positive nuclei in epidermal layers. *Panels* *3*, *4* allogeneic ASC wounds display several PCNA-positive cells. *Panels* *5*, *6* in wounds treated with BM-MSCs the number of PCNA-positive cells is low. *Panels* *7*, *8* saline-treated wounds show no epithelial cells marked with PCNA, but a considerable immunopositivity for fibroblasts and endothelial cells was detected. Eschar (*asterisk*). Immunostaining of PCNA. *Scale bar* 200 μm; original mag. ×100.

Finally, the number of PCNA-positive nuclei in the epidermal basal layers of autologous ASC-treated wounds (Figure 6, Panels 1 and 2) was higher than in all other groups (Figure 6, Panels 7 and 8). The saline-treated wounds, however, displayed increased positivity for fibroblasts and endothelial cells.

## Discussion

Skin is the largest organ of the human body and it has multiple functions in maintaining homeostasis. It forms an important barrier from the outside environment. Following skin injury, the damaged tissue is repaired through coordinated biological actions that constitute the cutaneous healing response. [1–4, 28].

Chronic wounds are an important and growing problem with an incidence of 5–7 million cases per person/years in the United States; and, about 50% do not respond to current treatment [6, 29, 30]. Moreover, skin grafting in children remains a challenge for pediatric surgeons and scientists.

The presence of MSCs in normal skin [31], their capacity to differentiate and to regulate immune and inflammatory responses suggest their role in wound healing [32–34]. For this reason the application of exogenous MSCs is a promising approach to treat non healing wounds.

Although BM-MSCs have been extensively used for cell therapy approaches in seminal experimental and clinical studies, adipose tissue has been increasingly employed as an abundant source of ASCs, especially in the field of reparative medicine [35]. Successful isolation, extensive proliferative capacities ex vivo, and their ability to secrete pro-angiogenic growth factors make adipose derived stem cells an ideal cell type for the treatment of non healing wounds [36–40], also in pediatric patients.

Topically delivered ASC have been shown to enhance granulation tissue formation in skin wounds improving outcome in wound healing. Skin wounds treated with ASC are characterized by an enhanced healing rate and less scar formation than control wounds [34].

It has been reported that the reparative effect of MSCs is mediated by paracrine signaling with release of biologically active molecules affecting cell migration, proliferation, and survival of the surrounding cells [1, 5, 6, 34]. Recent studies have documented that extracellular vesicles are a key component of paracrine secretion in many cell types, also including MSC [41–44].

MSCs contribute to the proliferative phase of wound healing and promote granulation and epithelialization by expressing growth factors such as VEGF, βFGF, and KGF [34]. Nakagawa et al. [45] suggested that MSCs, together with βFGF in a skin defect model, accelerate wound healing by showing that human MSCs differentiated into epithelium in a rat model. Shumakov et al. [46] observed that MSC transplantation onto deep burn wound surfaces decreased inflammatory cell infiltration and accelerated the formation of new vessels and granulation tissue in rats.

Results from our histological analysis are in agreement with data reported in the literature [47, 48] and show differences in the wound healing progress among experimental groups (different settings and sources). Improved re-epithelization, reduced inflammatory infiltration and increased collagen deposition were observed in biopsies from wounds treated with allogeneic or autologous ASCs compared to autologous BM-MSCs and control-wounds. In autologous ASC-treated wounds increased cellular proliferation was also noted. ASC inoculation provided more rapid wound closure than BM-MSCs and controls. It is well known that excessive collagen accumulation may induce hypertrophic scarring [4]: interestingly, areas treated with autologous ASCs displayed a collagen fiber pattern similar to normal skin in shape, size and orientation, thus suggesting that ASCs improve restoration of skin architecture during wound healing.

The ability of MSCs to promote the transition from inflammatory to the proliferative phase is particularly critical for treating wounds where high levels of inflammation prevent healing [12].

In the present study, earlier re-epithelialization, replacement of the fibrin clot by granulated tissue and collagen deposition in autologous ASC-treated wounds were observed. The unsatisfactory results observed with BM-MSCs does not seem to be related to the lower number of inoculated cells. The data confirm that even though MSCs from different tissues have similar levels of surface antigen expression and differentiation ability, their rate of cell proliferation may be different and MSCs from various sources may have different therapeutic potentials [16].

The possible oncogenic transformation during in vitro expansion remains a critical consideration in MSC in vivo applications. While human MSCs are apparently resistant to transformation, animal MSCs, in particular mouse MSCs, are prone to acquire genomic abnormalities [49–51]. In order to exclude this possibility, in the present study, we used cells at early passages of in vitro expansion. Moreover, cell growth in a semi-solid medium and capability to reach senescence were evaluated, showing that ASCs and BM-MSCs expanded from rabbits were not transformed.

Wound healing in the pediatric patient is of utmost clinical and social importance, since scarring can have aesthetic and psychological sequelae, from infancy to late adolescence. Despite the extensive data supporting the promising use of MSCs in dermal wound healing, clinical translation to the pediatric age remains limited [6, 52]. These data, from our preclinical study using young animals, could also be translated to regenerative medicine for the treatment of congenital and acquired skin lesions occurring in the pediatric age. At certain periods during childhood, such as in neonates or young infants, adipose tissue harvesting may be difficult or limited. Therefore, further studies focusing on the potential efficacy of cell therapy approaches in this particular area with the use of allogeneic ASCs obtained also from adult donors or cell free extracellular vesicles are urgently needed. Moreover, pre-clinical studies are necessary to validate the best skin regeneration technique, which would be used in translational pediatric surgery research in infants with disfiguring lesions.

## Conclusions

Rabbit ASC can be isolated and expanded in vitro with relative abundance. Topical inoculation of ASCs provides restoration of skin architecture during cutaneous wound healing. The use of ASCs could be a promising solution to treat non healing or extended wounds; and would be applicable also in children. Future experimental studies should provide further perspectives on regenerative medicine in pediatric surgery.

## Abbreviations

AP: alkaline phosphatase
ASCs: adipose stem cells
BM-MSCs: bone marrow-mesenchymal stromal cells
DAB: 3,3′-diaminobenzidine
DMEM: Dulbecco’s modified eagle medium
FBS: fetal bovine serum
FITC: fluorescein isothiocyanate
H&E: haematoxylin and eosin
MNC: mononuclear cells
MSCs: mesenchymal stromal cells
PBS: phosphate-buffered saline
PCNA: proliferating cell nuclear antigen
PE: phycoerythrin
SA-β-gal: β-galactosidase
SVF: stromal vascular fraction
